# Optimization of a Multiresidue Analysis of 65 Pesticides in Surface Water Using Solid-Phase Extraction by LC-MS/MS

**DOI:** 10.3390/molecules26216627

**Published:** 2021-11-01

**Authors:** Bahar Nakhjavan, Jason Bland, Maryam Khosravifard

**Affiliations:** Center for Analytical Chemistry, California Department of Food and Agriculture, Sacramento, CA 95832, USA; jason.bland@cdfa.ca.gov (J.B.); Maryam.khosravifard@cdfa.ca.gov (M.K.)

**Keywords:** solid-phase extraction, pesticides, LC-MS/MS

## Abstract

An analytical method was developed and validated for simultaneous quantitation of 65 pesticides, including one single solid-phase extraction (SPE) procedure in surface water by liquid chromatography coupled to tandem mass spectroscopy. Different parameters that have an influence on extraction efficiency were evaluated in this research. Different types of cartridges, elution solvents, and sorbent drying time were investigated, and the most appropriate one was selected. Moreover, various pretreatment techniques were applied to remove sediments from water without the loss of pesticides. Centrifugation was introduced as the best option at the beginning of sample preparation to resolve the clogging of the sorbent cartridges. The recoveries of all pesticides ranged from 70% to 120%, with a relative standard deviation of less than 13.7%. The feasibility of the method was evaluated on 10 surface water samples with different concentrations of sand, sediment, and particles.

## 1. Introduction

In the past decades, concern regarding the ecological influence of organic contaminants in water matrices has been enhanced. The mass production of these chemicals has led to their widespread and sometimes incorrect applications in agricultural industries resulting in their classification as toxic environmental contaminants [1]. The potential adverse effects of these pesticide residues on the water ecosystem have received significant attention.

Different national governments have set guidelines and procedures for the regulation of these chemicals in waters. Therefore, controlling them through monitoring is important to improve, protect and prevent further deterioration of water quality [2,3].

Several extraction techniques and analytical methods were developed and improved to address the trace analysis of residues. In preparing the sample, an effective clean-up procedure is required prior to instrument analysis [4,5]. This eliminates interferences (sediments, particles, organic compounds, biomolecules, pigments, etc.) present in water with low concentrations of pesticides.

Liquid-liquid extraction (LLE) and solid-phase extraction (SPE) are the most common sample preparation techniques used as a purification and clean-up process prior to instrumental analysis [6,7]. Solid-phase extraction (SPE) is gaining much interest as it includes different simultaneous mechanisms such as adsorption, partition, and ionic exchange with less organic solvent [8]. Due to this popularity, sample preparation using SPE has increased intensely; debate on appropriate SPE sorbent and extraction conditions that results in high extraction efficiencies for multiclass of pesticides continues [9,10].

Gas chromatography (GC) and liquid chromatography (LC) followed by tandem mass spectrometry are the most used analytical instruments recommended for the determination of a large number of compounds at trace levels [11,12,13]. Due to the high throughput, selectivity, and sensitivity for a wide range of pesticides, liquid chromatography coupled by tandem mass spectrometry (LC-MS/MS) is the most appropriate analytical instrumentation [14]. Additionally, numerous studies have verified that the type and design of the ionization source have a significant impact on the performance of the mass spectroscopy [15,16].

Linearity, accuracy, precision, standard deviation, and relative standard deviation were performed as part of a validation study as well as a storage stability study. Moreover, the solid-phase extraction method was applied to 10 surface water samples to confirm that this optimized SPE method is a superior choice for the simultaneous enrichment and purification of multiclass pesticides in a shorter time relative to traditional methods. The purpose of this study is (i) to develop one single SPE procedure for the simultaneous quantification of 65 multiclass pesticides in surface water for LC-MS/MS analysis, (ii) to validate this analytical method, and (iii) to check the feasibility of the new SPE on real surface water samples without loss of pesticide recoveries.

## 2. Results and Discussion

### 2.1. Optimization of LC-MS/MS Conditions

The optimized analysis was performed in the multiple reaction monitoring (MRM) based on the two most abundant transitions for quantification and confirmation. These two abundances and acquisition parameters were determined by the infusion of individual standard solutions into the tandem mass spectrometer. Analysis of pesticides was performed in positive mode, except for fipronil and its metabolites, which were analyzed in negative mode. MRM transitions, collision energy (CE), de-clustering potential (DP) as well as retention time (RT) are summarized in Table 1. The method detection limit (MDL) of 65 pesticides was estimated to assess the sensitivity of the instrument.

### 2.2. Optimization of the SPE Method

The development of an LC-MS/MS method for the quantification of different pesticides with different physicochemical properties can be a complex study because numerous parameters are involved. Therefore, optimizing an SPE method in one analysis covering a wide range of agriculturally used pesticides with different polarities is always desired.

In this research, some parameters, including sorbent type, elution solvent, and drying time, were studied to achieve the most appropriate extraction condition. Then, a unique solid-phase extraction was chosen and optimized to increase the extraction efficiency for a wide range of insecticides, herbicides, fungicides, and their metabolites.

#### 2.2.1. Sorbent Type

**Figure 1:**
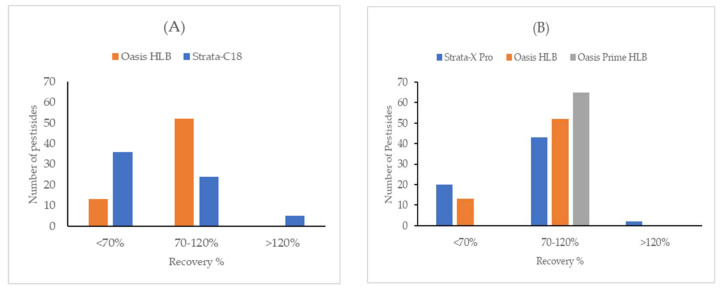
Comparison of recoveries for two main polymeric and silica-based sorbents (**A**) and three different polymeric SPE cartridges (**B**).

A broad variety of analytes (0.8 < log K_ow_ < 7.0) makes the sorbent selection challenging in this experiment. Two different types of the most used SPE cartridge were applied: Polymeric sorbent and silica-based end-capped C18. Both sorbents are the most recognized and have been used extensively in water chemistry [17,18]. It was confirmed that polystyrene-divinylbenzene-*N*-vinylpyrrolidone copolymers have a better potential than a silica-based sorbent for the extraction of the target pesticides from environmental water samples. As shown in Figure 1A, a great number of pesticides can be well recovered on polymeric sorbent (Oasis HLB, 500 mg) in comparison with bonded phase silica sorbent (Strata-C18, 500 mg) using a small sample volume of 100 mL. As a result, this sorbent type was chosen for the optimization. These outcomes are consistent with Donato et al. [9].

For this comparison, blank surface water samples were spiked with a mixed standard solution and isotopically labeled standards. It was subsequently enriched, eluted, and concentrated for LC-MS/MS analysis. As shown in Figure 1B, Strata X Pro, Oasis HLB, and Oasis PRiME HLB cartridges were compared. Oasis PRiME HLB was selected as the best candidate due to the high retention of the analytes of interest. Moreover, Oasis PRiME HLB demonstrates better recovery results by removing more matrix interferences such as salts, proteins, and phospholipids and was considered for further optimization in this work.

#### 2.2.2. Eluent

The significant role of elution solvent for desorption of analytes from the stationary phase is undeniable. Thus, the selection of an appropriate elution fraction for a large polarity range of analytes has attracted interest from researchers [19,20]. To achieve satisfactory recovery, several elution solvents and ratios were evaluated in this research.

MeOH/MTBE (10/90)(A), MeOH/DCM/MTBE (10/10/80)(B), MeOH/DCM (50/50)(C), ACE/DCM (50/50)(D), and the mix of EA/*n*-hexane/ACE (31/38/31) and MeOH (E) were examined to obtain high eluent efficiency. The mix of EA/*n*-hexane/ACE and MeOH with different polarities showed more eluent efficiencies among these mixed solvents for Oasis PRiME HLB with an average efficiency of 98.6%. The combination of EA/*n*-hexane/ACE with moderately polar/non-polar/semi-polar characteristics and polar methanol was designated as an efficient eluent. The recoveries for other elution combinations were lower, with averages of 78% for A, 63% for B, 59% for C, and 51% for D.

The solvent selection for analyte elution is a critical factor that has a significant influence on the retention and adsorption capability of sorbents toward target compounds. With the optimized elution solvents, no solvent exchange is required for the final solution. After concentrating the sample, the two were combined and injected directly to LC-MS/MS.

#### 2.2.3. Sorbent Drying Time

In order to achieve a satisfactory extraction procedure, sorbent drying was investigated as a critical step to avoid poor retention for target analytes. Lower recoveries were obtained when different drying times (from 0, 10, 30, and 60 min) were investigated. The eliminated or shortened drying time makes the final solution less concentrated and results in a reduction in sensitivity. If the residual water were not fully removed from the sorbent, then the remaining residual water can dilute the final elution. The optimized time of drying applied was one hour under high vacuum.

#### 2.2.4. Pretreatment

Despite the numerous advantages of SPE over LLE, clogging is the most problematic aspect of an SPE cartridge in samples containing suspended solids [21,22]. It can become quickly overloaded and clog the top frit before the sample ever reaches the sorbent. To overcome this issue when handling samples with high particulate levels, different alternatives have been advised. Filtration, glass wool, and double cartridges are recommended solutions that are widely used as a remedy in studies of clogging [23,24,25]. In this study, centrifugation was found to be the best technique to separate out sediment from the surface water sample to prevent blockage of the SPE.

Filtration is a convenient tool in preventing SPE clogging and reducing flow with particulate samples. Filter devices that have higher porosity filter out large particles before loading samples and allow the SPE bed to operate more efficiently. In this study, real surface water samples with different particulate concentrations were spiked and then added to the cartridges. In some cases, sorbents were blocked before entire samples were loaded, and other samples that had successful extractions were not recovered efficiently for two labeled standards. This experiment proved that filtration is not an appropriate clean-up technique to remove sediments and particles because membrane adsorption can reduce extraction efficiency.

Glass wool is a clean-up procedure for isolating impurities from aqueous samples before using solid-phase extraction media. To ensure that glass wool can be used as a physical barrier to line the cartridge, it was applied for real surface water samples. Not only was it observed that some particles do not become trapped by glass wool, but also the glass wool used will not be fully dry due to its spongy texture. The target compounds were not found at an acceptable recovery range and indicated that glass wool has poor control of the flow rate of water samples when passing through the cartridge pretreatment course.

Employing more than one cartridge in a series has recently received more attention. After spiking samples, two cartridges (Oasis PRiME HLB, 500 mg) were applied during the extraction procedure. This result displayed that a method consisting of two cartridges is an undesirable technique due to its time consuming, poor accuracy, inconsistency, remaining clogging issue, and cost.

In recent years, centrifugation has been used as an option to decrease drying time and reduce elution volume but not to prevent sediments depositing and clogging purpose in SPE cartridges [26,27]. Therefore, during this study, aqueous matrices were spiked and centrifuged, then the supernatant was loaded and extracted based on the described procedure in Section 3.4. The highest recoveries were observed when the centrifuging was used as a clean-up step, and as a result, it was chosen as the most appropriate clean-up procedure for surface water samples with different levels of suspended solids.

### 2.3. Application to Real Samples

After optimization, this method was used for 10 real surface water samples collected from different locations in California. The samples were collected in a 100 mL amber glass container and stored in the dark at 4 ± 4 °C. After spiking two isotopically labeled standards, they were centrifuged and extracted by the optimized SPE method described in this research. The presence of all positive pesticides was confirmed by deviation of precursor ions and retention time. All real samples were analyzed for 65 presides and 2 spiked labeled standards and showed different levels of pesticide residue. Labeled standard recoveries were between 72.6% and 105.2%, with a relative standard deviation between 7% and 13%. As shown in Table 2, this rapid and simple method investigated the presence of bensulide, chlorantraniliprole, chlothianidin, cyantraniliprole, dinotefuran, diuron, imidacloprid, methomyl, thiamethoxam, fipronil, fipronil sulfone, and desufinyl fipronil in more than one-third of samples. This extraction approach was successfully applied for the routine analysis of all aquaculture waters with various levels of particulates.

## 3. Materials and Methods

### 3.1. Chemicals and Reagents

HPLC-grade acetonitrile (ACN), methanol (MeOH), ethyl acetate (EA), acetone (ACE), *n*-hexane, water, and formic acid were purchased from Fisher Scientific (Waltham, MA, USA). Ammonium formate was purchased from Sigma-Aldrich (St. Louis, MO, USA). Oasis PRiME HLB (500 mg/6 mL) and Oasis HLB (500 mg/6 mL) were obtained from Waters (Maniford, MA, USA). Strata X Pro (500 mg/6 mL) and Strata C18-E (500 mg/6 mL) were purchased from Phenomenex (Torrance, CA, USA). All 65 standards were purchased from ChemService (West Chester, PA, USA).

### 3.2. Standards

Individual stock standards of 1.0 mg/mL were prepared in acetonitrile and stored in brown glassware. A combination standard of 10 µg/mL, including two surrogates, was prepared from the individual standards in acetonitrile. The combination standard was also used to dilute to the following concentrations: 0.00125, 0.0025, 0.005, 0.0125, 0.025, 0.05, 0.125, 0.25, 0.5 and 1 μg/mL in acetonitrile. These standards were then diluted in half with water right before use to make the following concentrations: 0.000625, 0.00125, 0.0025, 0.005, 0.0125, 0.025, 0.05, 0.125 μg/mL for instrument calibration. All these standard solutions were stored at −15 ± 5 °C.

### 3.3. Samples

The water samples used for the method development were taken from Lake Clementine in California. Real surface water samples were collected from different aquaculture locations within California state and stored in a refrigerator at 4 ± 4 °C prior to the sample preparation procedure.

### 3.4. Sample Preparation

Samples were removed from the refrigerator and allowed to reach ambient temperature. A volume of 100 mL of each sample was transferred into a centrifuge tube and centrifuged at 4000 rpm for 20 min. Samples were then ready for solid-phase extraction.

Cartridges were conditioned with 5 mL ethyl acetate, 5 mL *n*-hexane, 5 mL acetone, 5 mL methanol, and 5 mL water sequentially in a slow dropwise fashion. The samples were loaded to the columns and allowed to flow through 2–3 mL/min. A total of 5 mL HPLC-grade water was added and dried under vacuum for 60 min. In the next step, the cartridges were eluted with 6 mL of *n*-hexane-acetone (3:1) and 5 mL methanol separately. Two eluates were evaporated to 0.5 mL under nitrogen at 40 ± 5 °C and combined into 1 mL final volume for analysis.

### 3.5. Liquid Chromatography-Tandem Mass Spectrometry

The liquid chromatography and MS/MS optimization were studied in this research to find the most appropriate operating conditions by individual injection of each standard.

#### 3.5.1. Liquid Chromatography Separation Conditions

A Shimadzu LC30 liquid chromatograph equipped with Waters Acquity BEH C18 column (1.7 μm, 2.1 × 100 mm) was used. Samples were eluted using a gradient system at a flow rate of 0.4 mL/min throughout the 18 min run-time at 50 °C with an injection volume of 3 µL.

The mobile phases consisted of: (mobile phase A) 9 mM ammonium formate and 0.1% formic acid in 94:5 H_2_O/MeOH, and (mobile phase B) 9 mM ammonium formate and 0.1% formic acid in 90:9 MeOH/H_2_O. The gradient conditions were optimized as follows: 2% B from 0.01 to 0.25 min, 10–100% B from 0.25 to 10 min, 100% from 10 to 15 min, 100–2% from 15 to 15.10, and 0% B from 15.10 to 18 min.

#### 3.5.2. Mass Spectroscopy Conditions

To achieve a mass spectrum of the pesticides, a Triple Quad 6500 ABSciex mass spectrometer with a positive and negative ESI interface was used. Mass spectrometer operating parameters are summarized as follows: curtain gas: 20 psig, ion spray voltage: ±4500, temperature: 250 °C, ion source gas 1: 50, ion source gas 2: 50, collision gas: 8, MRM detection window: 30 and 60 s and target scan time: 0.2 and 0.4 for positive and negative mode, respectively. The analytes were monitored and quantified using multiple reaction monitoring (MRM). Sciex Analyst software 1.7 and Multiquant software 3.0 were applied for data acquisition and data processing, respectively.

### 3.6. Method Validation

The proposed method was validated by an in-house quality control procedure. Instrumental linearity, method detection limit, reporting limit, accuracy, and precision were achieved.

#### 3.6.1. Instrumental Linearity

A quadratic regression of the calibration data with all levels was used with weighted 1/x. The correlation coefficient (R^2^) was higher than 0.995 in all cases. The linearity was evaluated based on using eight-point calibration curves. The upper limit of linearity was set at 125 ng/mL as the highest concentration in the calibration curve.

#### 3.6.2. Method Detection and Reporting Limit

The method detection limit (MDL) refers to the lowest concentration of the analyte that a method can detect reliably. To determine the MDL, seven clean background surface water samples were spiked at 0.01 ng/mL for each analyte and processed through the entire method along with a blank. The standard deviation (SD) derived from the spiked sample recoveries was used to calculate the MDL using this equation:

MDL = t × SD (n = 7 replicates, t = 3.143)(1)

Reporting limit refers to a level at which reliable quantitative results may be obtained. The MDL is used as a guide to determine the RL. The RL is two times the MDL in this work. The calculated SD and RL for all pesticides are shown in Table 3.

#### 3.6.3. Accuracy and Precision

The method validation consisted of five sample sets, and each set included three levels of fortification. All spikes were processed through the entire analytical method. Spike levels, recoveries, standard deviation, and relative standard deviation for the target compounds are shown in Table 4. The recoveries were within the range of 70–120% for 65 multiresidue pesticides and 2 isotopically labeled standards. The reproducibility was measured by five days fortification study of 0.02, 0.05, and 0.1 ng/mL for each pesticide spiked to background surface water samples. The relative standard deviation (RSDs, n = 5) of recoveries was less than 14% for all pesticides. 

## 4. Conclusions

A total of 65 pesticides from different chemical classes, including organophosphates, carbamates, triazines, neonicotinoids, pyrethroids, pyrimidines, and others, were extracted efficiently in environmental waters. The proposed optimized extraction method demonstrates the practical applications in terms of easy sample pretreatment, reduction in organic solvent, short procedure time, small sample volume, and suitable recoveries. The final method was then validated at three different concentrations over five days. All pesticides were recovered at the range of 70–120% at these spiked levels, and the relative standard deviations (RSDs) for these analytes were estimated less than 14%. As a result, the developed method allows our analysts to provide the results with suitable reproducibility and high accuracy for the determination of target pesticides with no matrix effect.

## Figures and Tables

**Table 1:** MRM parameters for pesticides detection in positive and negative modes.

Compound	RT (min)	Precursor Ion	Quantification Transition (*m*/*z*)	DP (eV)	CE (eV)	Confirmatory Transition (*m*/*z*)	DP (eV)	CE (eV)
			**Positive Mode**					
Acetamiprid	3.19	223.0	125.9	56	25	89.9	56	43
Atazine	6.00	216.0	174.0	61	25	103.9	61	37
Azoxystrobin	7.10	404.0	371.9	41	19	344.0	41	33
Bensulide	8.57	397.9	313.8	31	15	157.9	31	31
Boscalid	7.38	342.9	306.9	101	25	270.9	101	43
Bromacil	4.88	260.9	204.9	26	17	187.8	26	37
Carbaryl	5.35	202.0	145.0	31	13	127.0	31	37
Chlorantraniliprole	6.70	483.9	285.8	51	17	452.9	51	21
Chlorsulfuron	5.32	357.9	141.0	106	21	167.1	106	21
Chlorpyrifos	10.46	349.8	197.9	41	25	96.8	41	45
Chlothianidin	2.86	249.9	168.9	36	15	131.9	36	19
Cyprodinil	8.28	226.0	93.0	81	41	108.1	81	35
Cyantraniliprole	5.68	473.0	284.0	76	17	442.0	76	29
Diazinon	8.89	305.0	169.0	66	29	153.0	66	27
Dichlorvos	4.87	220.9	108.9	86	23	78.9	86	34
3,4-dichloroaniline	7.00	161.9	127.0	140	30	109.0	140	40
Diflubenzuron	8.37	310.7	157.9	41	17	140.8	41	41
Dimethoate	3.14	229.9	198.9	21	13	124.8	21	27
Dinotefuran	2.03	203.0	129.1	43	14	114.0	43	16
Dithiopyr	9.79	402.0	354.0	116	23	272.0	116	37
Diuron	6.22	232.9	72.0	46	21	46.1	46	35
Ethoprophos	8.10	242.9	173.0	61	19	130.7	61	27
Fenamidone	7.30	311.9	236.0	56	19	92.0	56	39
Fenhexamid	8.03	301.9	97.1	21	29	54.9	21	59
Fludioxonil	7.33	265.9	229.0	20	13	158.0	20	43
Flupyradifurone	3.22	289.0	126.1	105	27	90.0	105	58
Hexazinone	4.94	253.1	171.0	41	21	71.0	41	39
Imidacloprid	2.80	256.0	208.9	30	21	175.1	30	25
Indoxacarb	9.70	527.8	150.1	76	27	202.9	76	49
Isoxaben	7.55	333.1	165.0	41	23	107.0	41	79
Kresoxim-Methyl	8.63	331.1	314.0	24	7	206.0	24	13
Linuron	6.96	249.0	182.0	51	23	160.0	51	25
Malathion	7.49	330.9	126.9	31	17	284.9	31	11
Mefenoxam	6.29	280.0	220.0	21	19	248.0	21	13
Methidathion	6.39	319.8	302.9	6	9	144.9	6	17
Methomyl	2.05	163.0	87.8	11	13	105.9	11	13
Methoxyfenozide	7.62	369.1	149.0	36	21	313.1	36	11
Metribuzin	4.81	215.1	187.0	36	25	84.0	36	31
Norflurazon	6.39	303.9	284.0	101	31	159.9	101	39
Oryzalin	8.22	347.0	304.9	41	19	288.0	41	23
Oxadiazon	10.31	344.9	302.9	91	17	219.9	91	27
Prometon	5.96	226.1	142.0	56	31	184.1	56	25
Prometryn	7.48	242.0	157.9	45	31	200	45	25
Propanil	6.99	217.9	161.9	56	21	126.9	56	33
Propargite	10.82	368.1	231.1	21	13	175.0	21	21
Propiconazole	8.93	341.9	158.9	56	31	69.0	56	23
Pyraclostrobin	9.08	388.0	193.9	36	17	163.0	36	31
Pyriproxyfen	10.34	322.0	95.9	46	19	184.9	46	29
Quinoxyfen	10.43	307.8	196.8	121	43	161.9	121	47
Simazine	4.88	202.0	124.0	61	25	103.9	61	33
*S*-Metolachlor	8.20	284.0	252.0	41	19	176.1	41	35
Sulfoxaflor	3.42	278.1	154.1	66	13	154.1	66	38
Tebuconazole	8.79	308.0	69.9	66	57	124.9	66	39
Tebufenozide	8.49	353.1	133.0	26	23	297.1	26	11
Tebuthiuron	5.05	229.0	172.0	46	23	115.8	46	35
Thiacloprid	3.59	252.9	125.8	76	27	90.0	76	51
Thiamethoxam	2.15	291.9	211.0	41	19	180.9	41	29
Thiobencarb	9.25	258.0	124.9	41	23	89.0	41	65
Trifloxystrobin	9.69	409.0	186.0	41	23	144.9	41	57
Atrazine-d5 (Surrogate)	5.94	220.9	179.0	61	25	101.0	61	31
Imidacloprid-d4 (Surrogate)	2.77	259.9	213.0	170	46	179.0	170	46
			**Negative Mode**					
Fipronil	8.55	436.8	329.8	−45	−22	331.8	−45	−22
Fipronil Amide	6.62	452.7	347.9	−25	−20	303.9	−25	−32
Fipronil Sulfide	8.75	418.8	382.8	−20	−18	261.5	−20	−38
Fipronil Sulfone	9.01	450.8	414.9	−40	−22	281.8	−40	−36
Desulfinyl Fipronil	8.35	387.0	350.9	−45	−16	281.8	−45	−42
Desulfinyl Fipronil Amide	5.61	405.0	369.0	−50	−12	329.0	−50	−30

Retention time (RT); de-clustering potential (DP); collision energy (CE).

**Table 2:** 10 surface water samples were measured for all pesticides with this optimized SPE method.

Analyte (ng/mL)	SW1	SW2	SW3	SW4	SW5	SW6	SW7	SW8	SW9	SW10
Acetamiprid	ND	ND	ND	ND	ND	ND	Trace	0.0977	0.123	Trace
Atrazine	ND	ND	ND	ND	ND	ND	ND	ND	ND	ND
Azoxystrobin	Trace	Trace	ND	Trace	Trace	Trace	0.158	0.0338	Trace	Trace
Bensulide	ND	ND	ND	Trace	ND	ND	0.9710	7.53	52.3	0.083
Boscalid	ND	ND	ND	Trace	ND	ND	0.852	1.27	0.565	Trace
Bromacil	Trace	ND	ND	Trace	ND	ND	ND	ND	ND	ND
Carbaryl	ND	Trace	ND	Trace	ND	ND	ND	ND	ND	ND
Chlorantraniliprole	Trace	ND	ND	Trace	Trace	0.0205	0.062	0.229	0.515	Trace
Chlorsulfuron	ND	ND	ND	ND	ND	ND	ND	ND	ND	ND
Chlorpyrifos	ND	ND	ND	ND	ND	ND	ND	ND	ND	ND
Clothianidin	Trace	0.0228	0.0318	Trace	Trace	Trace	0.0956	0.90	0.550	Trace
Cyprodinil	ND	ND	ND	ND	ND	ND	Trace	ND	0.103	ND
Cyantraniliprole	ND	ND	ND	ND	ND	ND	0.111	0.517	0.164	0.0568
Diazinon	ND	ND	ND	ND	ND	ND	ND	ND	ND	ND
Dichlorvos	ND	ND	ND	ND	ND	ND	ND	ND	ND	ND
3,4-dicloroaniline	ND	ND	ND	ND	ND	ND	ND	ND	ND	ND
Diflubenzuron	ND	ND	ND	ND	ND	ND	ND	ND	ND	ND
Dimethoate	ND	ND	ND	ND	ND	ND	ND	ND	ND	ND
Dinotefuran	0.0478	0.0262	0.0212	0.041	0.0371	Trace	ND	ND	ND	ND
Dithiopyr	Trace	0.0227	0.0252	Trace	Trace	0.0326	ND	ND	ND	ND
Diuron	0.0264	0.128	0.0721	Trace	Trace	0.0252	ND	ND	ND	ND
Ethoprop	ND	ND	ND	ND	ND	ND	ND	ND	ND	ND
Fenamidone	ND	ND	ND	ND	ND	ND	Trace	Trace	Trace	ND
Fenhexamid	ND	ND	ND	ND	ND	ND	ND	ND	ND	ND
Fludioxonil	ND	ND	ND	ND	ND	ND	0.0143	ND	0.0664	ND
Flupyradifurone	ND	ND	ND	ND	ND	ND	0.0865	0.115	0.274	ND
Hexazinone	ND	ND	ND	ND	ND	ND	ND	ND	ND	ND
Imidacloprid	0.0397	0.0463	0.0274	0.263	0.0300	0.0256	0.148	0.254	0.159	ND
Indoxacarb	ND	ND	ND	ND	ND	ND	ND	ND	ND	ND
Isoxaben	ND	ND	ND	ND	ND	Trace	ND	ND	ND	ND
Kresoxim-Methyl	Trace	Trace	Trace	Trace	Trace	ND	ND	0.0292	0.296	ND
Linuron	ND	ND	ND	ND	ND	ND	ND	ND	ND	ND
Malathion	ND	ND	ND	ND	ND	ND	ND	ND	ND	ND
Mefenoxam	ND	ND	ND	ND	ND	ND	0.0299	0.0245	0.0207	ND
Methidathion	ND	ND	ND	Trace	Trace	ND	ND	ND	ND	ND
Methomyl	ND	ND	ND	ND	ND	ND	0.30	0.399	3.43	0.0224
Methoxyfenozide	ND	ND	ND	Trace	ND	ND	Trace	0.0312	0.0333	ND
Metribuzin	ND	ND	ND	0.0292	ND	ND	ND	ND	ND	ND
Norflurazon	ND	ND	ND	ND	ND	ND	ND	ND	ND	ND
Oryzalin	Trace	Trace	ND	Trace	ND	ND	ND	ND	ND	ND
Oxadiazon	Trace	ND	ND	ND	Trace	Trace	ND	ND	ND	ND
Prometon	ND	ND	ND	ND	ND	ND	ND	ND	ND	ND
Prometryn	ND	ND	ND	ND	ND	ND	0.159	Trace	Trace	0.0493
Propanil	ND	ND	ND	ND	ND	ND	ND	ND	ND	ND
Propargite	ND	ND	ND	ND	ND	ND	ND	ND	ND	ND
Propiconazole	Trace	Trace	Trace	Trace	0.0278	Trace	0.0236	ND	Trace	ND
Pyraclostrobin	ND	ND	ND	ND	ND	ND	0.135	Trace	0.021	ND
Pyriproxyfen	ND	ND	ND	ND	ND	ND	ND	ND	ND	ND
Quinoxyfen	ND	ND	ND	ND	ND	ND	Trace	ND	Trace	ND
Simazine	ND	ND	ND	ND	ND	ND	Trace	ND	ND	ND
*S*-Metolachlor	ND	ND	ND	ND	ND	ND	ND	Trace	ND	ND
Sulfoxaflor	ND	ND	ND	ND	ND	ND	0.025	0.684	0.566	ND
Tebuconazole	0.0501	0.0719	Trace	0.0772	Trace	Trace	ND	ND	ND	ND
Tebufenozide	ND	ND	ND	ND	ND	ND	ND	ND	ND	ND
Tebuthiuron	ND	ND	ND	Trace	ND	ND	ND	ND	ND	ND
Thiacloprid	ND	ND	ND	ND	ND	ND	ND	ND	ND	ND
Thiamethoxam	Trace	0.0591	Trace	0.0207	0.0296	0.0575	0.0701	0.371	1.13	0.0221
Thiobencarb	ND	ND	ND	ND	ND	ND	ND	ND	ND	ND
Trifloxystrobin	ND	ND	ND	ND	ND	ND	ND	Trace	ND	ND
Atrazine-d5 (Surrogate)	0.0407	0.0406	0.0426	0.0405	0.0407	0.0412	0.05	0.0468	0.0425	0.0426
Imidacloprid-d4 (Surrogate)	0.0459	0.0412	0.0418	0.0376	0.0426	0.0415	0.054	0.0526	0.0363	0.0437
Fipronil	0.0273	0.0290	Trace	0.141	0.0803	0.0249	ND	ND	ND	ND
Fipronil Amide	Trace	Trace	Trace	0.022	Trace	Trace	ND	ND	ND	ND
Fipronil Sulfide	Trace	Trace	Trace	Trace	Trace	Trace	ND	ND	ND	ND
Fipronil Sulfone	0.0383	0.0345	0.0836	0.110	0.0846	0.0398	ND	ND	ND	ND
Desulfinyl Fipronil	Trace	0.0238	Trace	0.0957	0.074	0.0214	ND	ND	ND	ND
Desulfinyl Fipronil Amide	Trace	Trace	Trace	Trace	Trace	Trace	ND	ND	ND	ND

Trace is the value that falls within MDL and reporting limit. ND is defined as non-detect and refers to values that are less than MDL.

**Table 3:** RL study for 65 multiresidue pesticides and 2 isotopically labeled standards in water (n = 7) at 0.01 ng/mL.

Compound	SD (ng/mL)	RL (ng/mL)	Compound	SD (ng/mL)	RL (ng/mL)
Acetamiprid	0.00038	0.00238	Methidathion NH_4_	0.00026	0.00163
Atrazine	0.00042	0.00261	Methomyl	0.00026	0.00163
Azoxystrobin	0.00051	0.00318	Methoxyfenozide	0.00037	0.00231
Bensulide	0.00021	0.00133	Metribuzin	0.00065	0.00410
Boscalid	0.00055	0.00345	Norflurazon	0.00027	0.00169
Bromacil	0.00037	0.00230	Oryzalin	0.00101	0.00637
Carbaryl	0.00052	0.00329	Oxadiazon	0.00048	0.00300
Chlorantraniliprole	0.00041	0.00257	Prometon	0.00032	0.00200
Chlorsulfuron	0.00039	0.00246	Prometryn	0.00040	0.00253
Chlorpyrifos	0.00041	0.00257	Propanil	0.00058	0.00366
Clothianidin	0.00066	0.00417	propargite NH_4_	0.00028	0.00174
Cyprodinil	0.00027	0.00170	Propiconazole	0.00041	0.00255
Cyantraniliprole	0.00068	0.00428	Pyraclostrobin	0.00031	0.00195
Diazinon	0.00055	0.00344	Pyriproxyfen	0.00026	0.00164
Dichlorvos	0.00038	0.00237	Quinoxyfen	0.00058	0.00366
3,4-dicloroaniline	0.00046	0.00289	Simazine	0.00024	0.00149
Diflubenzuron	0.00033	0.00208	*S*-Metolachlor	0.00041	0.00259
Dimethoate	0.00042	0.00264	Sulfoxaflor	0.00067	0.00419
Dinotefuran	0.00030	0.00187	Tebuconazole	0.00076	0.00475
Dithiopyr	0.00029	0.00180	Tebufenozide	0.00032	0.00202
Diuron	0.00020	0.00128	Tebuthiuron	0.00040	0.00250
Ethopropos	0.00050	0.00316	Thiacloprid	0.00051	0.00321
Fenamidone	0.00067	0.00420	Thiamethoxam	0.00054	0.00341
Fenhexamid	0.00082	0.00517	Thiobencarb	0.00042	0.00265
Fludioxonil NH_4_	0.00068	0.00429	Trifloxystrobin	0.00029	0.00180
Flupyradifurone	0.00036	0.00225	Atrazine-d5	0.00043	0.00273
Hexazinone	0.00045	0.00286	Imidacloprid-d4	0.00095	0.00594
Imidacloprid	0.00085	0.00537	Fipronil	0.00028	0.00175
Indoxacarb	0.00106	0.00667	Fipronil Amide	0.00088	0.00551
Isoxaben	0.00032	0.00201	Fipronil Sulfide	0.00066	0.00412
Kresoxim-Methyl NH_4_	0.00071	0.00446	Fipronil Sulfone	0.00025	0.00155
Linuron	0.00039	0.00247	Desulfinyl Fipronil	0.00033	0.00208
Malathion	0.00028	0.00177	Desulfinyl Fipronil Amide	0.00056	0.00352
Mefenoxam	0.00027	0.00167			

Reporting limit (RL); standard deviation (SD).

**Table 4:** Accuracy and precision of LC-MS/MS method for determination of 65 pesticides in surface water (n = 5).

Analyte	Concentration (ng/mL)	Mean Recovery (%)	RSD (%)	Analyte	Concentration (ng/mL)	Mean Recovery (%)	RSD (%)	Analyte	Concentration (ng/mL)	Mean Recovery (%)	RSD (%)
Acetamipirid	0.02	104.7	2.8	Cyprodinil	0.02	104.8	7.5	Fenamidone	0.02	101.1	4.5
	0.05	109.2	3.8		0.05	106.9	4.4		0.05	105.6	6.6
	0.1	105.6	7.6		0.1	105.1	7.3		0.1	106.9	3.9
Atrazine	0.02	104.5	2.7	Cyantraniliprole	0.02	92.9	3.0	Fenhexamid	0.02	89.5	10.2
	0.05	110.0	3.6		0.05	95.9	12.7		0.05	89.1	11.8
	0.1	110.2	5.5		0.1	87.8	11.7		0.1	83.6	5.9
Azoxystrobin	0.02	103.4	3.0	Diazinon	0.02	94.9	4.9	Fludioxonil	0.02	89.9	4.7
	0.05	109.0	5.7		0.05	100.9	9.4		0.05	105.1	6.6
	0.1	105.8	7.1		0.1	101.6	7.8		0.1	104.4	5.2
Bensulide	0.02	96.1	5.6	Dichlorvos	0.02	81.1	7.2	Flupyradifurone	0.02	106.5	0.4
	0.05	100.7	8.3		0.05	82.2	14.3		0.05	111.5	3.9
	0.1	101.9	11.6		0.1	76.2	11.7		0.1	109.5	3.8
Boscalid	0.02	108.8	2.5	3,4-dichloroaniline	0.02	103.7	3.9	Hexazinone	0.02	98.5	8.5
	0.05	110.1	6.8		0.05	106.1	7.1		0.05	101.5	8.5
	0.1	109.4	6.3		0.1	106.2	3.7		0.1	102.2	4.2
Bromacil	0.02	91.3	9.6	Diflubenzuron	0.02	94.1	3.3	Imidacloprid	0.02	100.4	3.6
	0.05	94.6	9.6		0.05	95.5	11.3		0.05	105.2	5.4
	0.1	88.8	5.9		0.1	88.5	6.7		0.1	102.0	7.8
Carbaryl	0.02	96.3	7.5	Dimethoate	0.02	102.5	2.1	Indoxacarb	0.02	85.0	7.0
	0.05	108.4	3.0		0.05	106.9	4.5		0.05	90.5	5.8
	0.1	111.5	2.9		0.1	109.1	2.0		0.1	90.6	12.8
Chlorantraniliprole	0.02	99.5	5.8	Dinotefuran	0.02	97.1	2.5	Isoxaben	0.02	106.4	3.1
	0.05	98.2	10.8		0.05	101.6	5.2		0.05	111.3	2.8
	0.1	93.1	10.6		0.1	70.4	8.8		0.1	108.9	5.1
Chlorsulfuron	0.02	96.3	9.2	Dithiopyr	0.02	93.6	3.7	Kresoxim-Methyl NH_4_	0.02	82.3	5.4
	0.05	99.6	13.2		0.05	100.0	9.8		0.05	99.4	3.9
	0.1	91.1	5.7		0.1	100.3	10.5		0.1	100.0	7.1
Chlorpyrifos	0.02	78.1	6.9	Diuron	0.02	99.0	3.9	Linuron	0.02	105.1	1.7
	0.05	79.6	8.7		0.05	107.6	6.0		0.05	109.4	5.2
	0.1	77.5	8.5		0.1	108.5	4.6		0.1	107.5	5.4
Chlothianidin	0.02	89.9	5.8	Ethopropos	0.02	105.2	4.9	Malathion	0.02	100.4	3.4
	0.05	92.3	12.7		0.05	107.8	6.1		0.05	106.5	6.2
	0.1	89.1	8.0		0.1	109.9	3.4		0.1	106.8	4.4
Mefenoxam	0.02	106.9	2.4	Propiconazole	0.02	98.0	1.9	Thiobencarb	0.02	89.6	5.0
	0.05	109.9	3.6		0.05	104.8	5.8		0.05	92.7	8.0
	0.1	109.6	3.8		0.1	104.0	5.9		0.1	95.0	7.9
Methidathion NH_4_	0.02	99.3	0.7	Pyraclostrobin	0.02	100.4	5.2	Trifloxystrobin	0.02	98.9	3.8
	0.05	108.3	5.4		0.05	102.6	7.8		0.05	101.5	4.6
	0.1	107.8	5.4		0.1	101.2	8.6		0.1	99.4	7.3
Methomyl	0.02	76.0	5.6	Pyriproxyfen	0.02	72.0	2.8	Atrzaine-d5	0.02	103.2	5.4
	0.05	81.2	7.0		0.05	72.0	5.1		0.05	109.3	4.3
	0.1	73.2	11.6		0.1	70.5	6.6		0.1	107.9	5.2
Methoxyfenozide	0.02	110.8	2.9	Quinoxyfen	0.02	72.6	3.6	Imidacloprid-d4	0.02	98.9	12.6
	0.05	113.3	1.8		0.05	73.5	6.2		0.05	102.3	9.5
	0.1	107.3	3.6		0.1	71.3	7.4		0.1	101.9	13.7
Metribuzin	0.02	101.2	1.4	Simazine	0.02	107.6	3.6	Fipronil	0.02	99.1	7.0
	0.05	108.8	4.0		0.05	110.4	3.9		0.05	106.1	8.4
	0.1	108.4	5.7		0.1	109.9	5.0		0.1	105.4	5.0
Norflurazon	0.02	103.8	1.5	*S*-Metolachlor	0.02	107.2	4.1	Fipronil Amide	0.02	106.5	3.4
	0.05	107.3	4.0		0.05	110.2	5.0		0.05	109.0	3.9
	0.1	107.9	4.9		0.1	109.9	4.8		0.1	109.3	7.6
Oryzalin	0.02	108.8	2.5	Sulfoxaflor	0.02	105.3	4.7	Fipronil Sulfide	0.02	99.4	5.2
	0.05	102.7	12.4		0.05	107.4	7.2		0.05	104.5	7.6
	0.1	102.9	6.7		0.1	106.1	6.5		0.1	102.6	6.1
Oxadiazon	0.02	94.5	3.0	Tebuconazole	0.02	101.1	6.2	Fipronil Sulfone	0.02	105.1	4.8
	0.05	98.2	5.9		0.05	106.4	7.4		0.05	107.6	4.3
	0.1	96.9	8.8		0.1	104.1	7.0		0.1	105.1	5.2
Prometon	0.02	110.8	2.6	Tebufenozide	0.02	107.0	2.3	Desulfinyl Fipronil	0.02	104.2	5.0
	0.05	112.9	4.1		0.05	109.1	3.7		0.05	108.9	5.0
	0.1	111.7	1.8		0.1	109.8	4.7		0.1	109.1	5.2
Prometryn	0.02	93.5	3.9	Tebuthiuron	0.02	105.7	4.0	Desulfinyl Fipronil Amide	0.02	104.4	4.8
	0.05	103.4	7.7		0.05	111.0	4.0		0.05	111.3	7.6
	0.1	105.2	6.1		0.1	108.9	3.6		0.1	109.2	5.1
Propanil	0.02	104.3	2.0	Thiacloprid	0.02	105.7	2.5				
	0.05	109.6	2.9		0.05	110.5	4.0				
	0.1	109.5	3.1		0.1	108.6	4.5				
Propargite NH_4_	0.02	76.4	6.5	Thiamethoxam	0.02	88.1	4.8				
	0.05	78.4	9.3		0.05	89.2	11.7				
	0.1	76.0	11.7		0.1	87.8	7.3				

Relative standard deviation (RSD).

## Data Availability

Not applicable.

## Sample Availability

Samples of the compounds are not available from the authors.
